# Growth Profile and Its Association with Nutrient Intake and Dietary Patterns among Children and Adolescents in Hail Region of Saudi Arabia

**DOI:** 10.1155/2017/5740851

**Published:** 2017-02-20

**Authors:** Eyad Alshammari, Epuru Suneetha, Mohd Adnan, Saif Khan, Awfa Alazzeh

**Affiliations:** Department of Clinical Nutrition, College of Applied Medical Sciences, University of Ha'il, P.O. Box 2440, Ha'il, Saudi Arabia

## Abstract

Hail region of Saudi Arabia (KSA) has the highest adult obesity rates in the entire kingdom and limited information is available about the prevalence and patterns of growth markers. Therefore, it is important to monitor the growth trends to implement effective public health preventive strategies for the region. This study aims to estimate the prevalence of growth profile patterns (stunting, wasting, underweight, and overweight) and its associations with nutrient intake and dietary patterns among children and adolescents. A cross-sectional survey was conducted involving 1420 children and adolescents (2–18 years), selected using a multistage stratified random-sampling technique representing both female and male schools from Hail region, KSA. Growth profile *z*-scores were generated using 2006 and 2007 WHO growth standards. The overall prevalence of 4.73% moderate and 1.54% severe underweight; 6.65% moderate and 2.59% severe stunting; 6.34% moderate and 2.55% severe wasting was present in the study population. Stunting decreased as age progressed with concurrent increase in the prevalence of overweight and obesity. There was a significantly higher prevalence of overweight (18.55% versus 23.05%; *P* < 0.001) and obesity (8.7% versus 13.85%; *P* < 0.001) in adolescents than in school-age children with higher prevalence in females as compared to males. Both stunted and overweight/obesity groups had significantly lower mean intakes for critical micronutrients necessary for growth as compared to normal children.

## 1. Introduction

Child growth assessment based on weight, height, and age in comparison to references and standards is internationally recognized as an important public health indicator for monitoring nutritional status and health in populations [1, 2]. Stunting (inadequate length/height for age) indicates early chronic exposure to undernutrition; wasting (inadequate weight for height) captures acute undernutrition; underweight (inadequate weight for age) is a composite indicator that includes elements of stunting and wasting [3]. Understanding the prevalence and patterns of long term growth falters like stunting along with the increasing advent of overweight/obesity in children and adolescents is crucial for any country's planning and implementation of public health policy. Although isolating the “nutrition factor” or the “child development factor” is complicated by the countless interactions of nutrition, nature, and nurture, research provides undoubted evidence of association between all degrees of malnutrition induced anthropometric deficits and morbidity and mortality [4, 5].

Rapid economic transition in KSA along with urbanization, globalization, and technological and social changes has resulted in nutrition transition. Nutrition transition referring to change in traditional diet patterns to western diet patterns which are high in energy and low in cost is often accompanied by changes in physical activity and other lifestyle factors. This in turn also undergoes a paradigm shift along with changing environmental exposures and availability of resources driven by increasing economic development. Living standards are on a rise, leading to complex changes in diet, lifestyle, and health patterns [6, 7]. Concurrent with this epidemiological transition trend, the prevalence rates of overweight and obesity are sharply rising in KSA [8].

Double burden of malnutrition is presenting a unique challenge to the world. In comparison to two decades ago, today there are 54 percent more overweight children globally and 35 percent fewer stunted children [9]. In a recent study, prevalence of malnutrition in Saudi children with other developed, transitional, and less developed countries was compared [10] and concluded that a huge disproportion still persist in the 21st century with about 40% prevalence of moderate underweight in Bangladesh and Yemen to 2.2% in Brazil and 1.3% in USA. They attributed this huge gap to the availability resources based on economic development. It also reported a prevalence of moderate underweight in Saudi children of 6.9% which is closer to the 5.4% reported from Egypt indicating an intermediate position, a 9.8% prevalence of wasting in Saudi children closer to 11.2% rates reported from Nigeria and a 10.9% prevalence of moderate stunting as compared to 12.9% reported from neighboring regional country like Oman [10]. Another study indicated concurrent national wide prevalence of overweight, obesity, and severe obesity in all age groups as 23.1%, 9.3%, and 2%, respectively, with boys having a significantly higher prevalence of obesity than girls [11]. In addition, further study indicated existing regional differences in prevalence of short stature, which varies sharply in various regions as compared to a national prevalence of 11% [12]. This emphasizes the need for regional studies to understand local environmental factors resulting in variation for growth parameters in children and adolescents. Efforts to combat “double burden” situation is therefore need of the hour which requires understanding current children and adolescent food and dietary patterns along with their interactions with other lifestyle and environmental exposures.

Hail region, the north of KSA, was reported to be having the highest percentage of obese adult population (33.9%) among all the regions of the KSA [8]. Coupled with it is a recent study which indicates a substantial prevalence of undernutrition among children and adolescents from Hail region [12]. The recent massive development of Hail into a fast growing suburban city from a traditional agrarian society emphasizes the pressing need for a thorough epidemiological survey in this region introspecting for lifestyle factors responsible for double burden of malnutrition from young ages. This exploration can help in identifying health risk behaviors which are specific to Hail region and will subsequently be advantageous in expanding preventive strategies which can help combat double burden of malnutrition in the region.

In view of above discussion, the current study aims to survey the prevalence of malnutrition in children (2–18 yr) and to estimate the prevalence of growth profile patterns (stunting, wasting, underweight, and overweight) and its associations with nutrient intake and dietary patterns in Hail region, KSA.

## 2. Materials and Methods

### 2.1. Study Sample and Settings

A cross-sectional survey was conducted during September 2015 to January 2016 using a multistage stratified random-sampling technique. A stratified random-sampling method was used after identifying nursery, primary, and secondary schools from urban and rural areas of Hail region. All secondary schools were unisex schools while nursery and primary schools were mostly mixed schools. Therefore, a total of 16 schools were selected randomly across the Hail region to represent urban and rural areas equally and stratified by age and sex. Further, classrooms were randomly selected from each selected school, all of which agreed to participate.

The study sample (age group 2–18 years) was selected from nursery, primary, and secondary schools (girls and boys) present in Hail region, Saudi Arabia. A total of 1670 students were surveyed through trained research assistants to collect anthropometric measurements of standing height and weight following all standard procedures. However, the final analysis included only 1420 samples after the exclusion of incomplete survey forms/questionnaire and nonreturned forms/questionnaire from parents. A core questionnaire was administered to identify sociodemographic information, developmental stage, dietary intake patterns, physical activity patterns, and other triggers such as specific exposures or hospitalization and medical history. 24 hr recall information was collected to estimate average nutrient intake.

### 2.2. Anthropometric Measurements

Anthropometric measurements such as body weight and height were measured using standard techniques. Body height was measured using stadiometer (DETECTO, USA) to the nearest 0.01 m, and body weight was measured with the subjects wearing light clothing and no shoes using mechanical flat scale (DETECTO, USA) to the nearest 0.1 kg. Body mass index (BMI) was calculated as the ratio of weight (kg) to the square of height (m).

### 2.3. Questionnaire

A questionnaire (see Supplementary Material, S1–S4, available online at https://doi.org/10.1155/2017/5740851) requesting information on demographic, socioeconomic information, medical history, food and dietary habits, physical activity, leisure activities, neighborhood opportunities, and developmental milestones was administered for all participants. Questionnaires were originally constructed in English and translated into Arabic. Forward and backward translation was done to crosscheck linguistic validity. An initial pilot was conducted for cultural validity among 30 samples including various stakeholders expected to participate in the study like parents from rural and urban areas and adolescents.

The questionnaires were filled with the help of parents up to 12-year-old children, whereas 13–18-year-old adolescents filled the questionnaires by themselves. Any help required for translation or extra information was provided by the research assistants. Schools were contacted a week before the data collection and class rooms were selected randomly representing different ages. A data collection room was priorly fixed and the selected class students were invited to participate in the survey for anthropometric measurements followed by comfortable seating arrangements for questionnaire filling.

### 2.4. Data Analysis

Weight for age *z*-scores (WAZ), height for age *z*-scores (HAZ), and weight for height *z*-scores (WHZ) for children up to 60 completed months were generated using the WHO Anthro, version 3.2.2, January 2011 [13]. *Z*-scores for those aged 5–18 years were determined using the WHO Anthroplus software [14]. For children 5–19 years the +1 SD in the WHO reference (equivalent to the 85th percentile) coincides at 19 years with the adults cut-off of BMI = 25 [kg/m^2^], which is the cut-off for overweight. Similarly, the +2 SD (equivalent to the 97th centile) coincides at 19 years with the adults cut-off of BMI = 30 [kg/m^2^], which is the recommended cut-off for obesity. Consequently the +3 SD cut-off was considered severely obese (corresponding to a BMI of above 35 [kg/m^2^]). For thinness and severe thinness the cut-offs are −2 and −3 SD, respectively.

The data set was cleaned and edited for inconsistencies. Missing data were not statistically computed. Statistical analyses were performed using the Statistical Package for Social Sciences (version 16.0, SPSS, Inc.) software. Patterns of the prevalence of stunting (HAZ < −2), underweight (WAZ < −2), wasting (WHZ < −2), overweight, and obesity by age and sex were determined. Descriptive statistics such as means and standard deviations were calculated for the continuous variables and frequencies for qualitative data. Student's *t*-test and chi-square analysis were used to examine differentials in variables. All reported *P* values were 2-sided and differences were considered statistically significant at *P* < 0.05.

## 3. Results and Discussion

A total of 1420 subjects (2–18 years) of which 700 male (49.3%) and 720 female (50.7%) subjects participated in the present survey. A total of 312 children were from 2 to <5 years' age group (30.0% of total subjects; 48.1% males and 51.9% females); 470 children were from 5 to 12 years' age group (33.1% of total subjects; 47.9% males and 52.1% females), while another 637 participants were from the age group 13–18 years (44.9% of total subjects; 51.0% males and 49.0% females).

Table 1 presents the prevalence of moderate (4.73%) and severe underweight (1.54%) and presents a nonlinear trend. A progressive increase in the prevalence of underweight with advancing age is noticed only from 2 to <5 years age cut-offs and later tapering on with increasing age. No statistically significant differences were observed between the two genders for percent prevalence rates.

Table 2 presents a prevalence of stunting of 6.65% and 2.59% for moderate and severe stunting, respectively. Stunting prevalence is higher as compared to underweight prevalence, indicating chronic malnutrition related issues in the study population. However, the trends of stunting were similar to underweight patterns, that is, progressively increasing through 2 to <5 years and tapering from there. Comparatively, females were having lower prevalence of stunting for most age groups; however, the differences for gender were statistically not significant.

Table 3 shows a prevalence of wasting of 6.34% and 2.55% for moderate and severe degrees, respectively. The overall prevalence was consistently lower in females and was found to be statistically significant for moderate wasting for males and females for the age groups 2 to <3 years (8.9 versus 6.8; *P* < 0.01) and 4 to <5 years (7.9 versus 5.9; *P* < 0.01). Other age group differences were not statistically significant.

The overall prevalence of overweight from 5 to 18 year age groups was 19.65% for boys and 21.95% for girls with a total of 20.8%. In addition, there was a significantly higher prevalence of overweight (18.55% versus 23.05%; *P* < 0.001) and obesity (8.7% versus 13.85%; *P* < 0.001) in adolescents than in school-age children. The prevalence of overweight and obesity in adolescents from 13 to 18 years of age was 20.8% and 11.27%, respectively. Females had a statistically significant higher prevalence of overweight and obesity for both school-age and adolescent age groups (Table 4).

The children dietary intakes were measured by 24 hr recall method. The mean daily nutrient intakes of the subjects of stunted and normal children and adolescents (2–18 years) were presented in Table 5 and mean daily nutrient intakes of the subjects of overweight/obese and normal children and adolescents (2–18 years) were presented in Table 6. Significant differences were found between stunted and normal children and adolescents for protein, calcium, potassium, and iron intakes (*P* < 0.05) and between overweight/obese and normal children and adolescents for energy, protein, calcium, and iron and potassium intakes (*P* < 0.05). Children and adolescents with short stature had significantly lower mean protein (37.3 ± 2.3 g) as compared to normal children and adolescents (45.6 ± 4.5 g). They also had significantly lower mean values for calcium and potassium which have a significant role in bone growth. Zinc and vitamin A, the nutrients known to play a significant role in growth and development processes, were also on lower side of the mean intakes as compared to normal children and adolescents. The differences were not statistically significant. However, overweight/obese children and adolescents had significantly higher energy, protein, and iron mean intakes as compared to normal children and adolescents. They also had significantly low mean levels for calcium and potassium. Overall mean intakes of B-complex vitamins for all groups are less than recommended dietary allowances indicating substantial poor quality of diets for micronutrient consumption. Noteworthy is the fact that the needed intake of micronutrients across the different ages was not investigated due to small sample size. This may change the overall statistical data about average daily nutrient intake.

**Figure 1:**
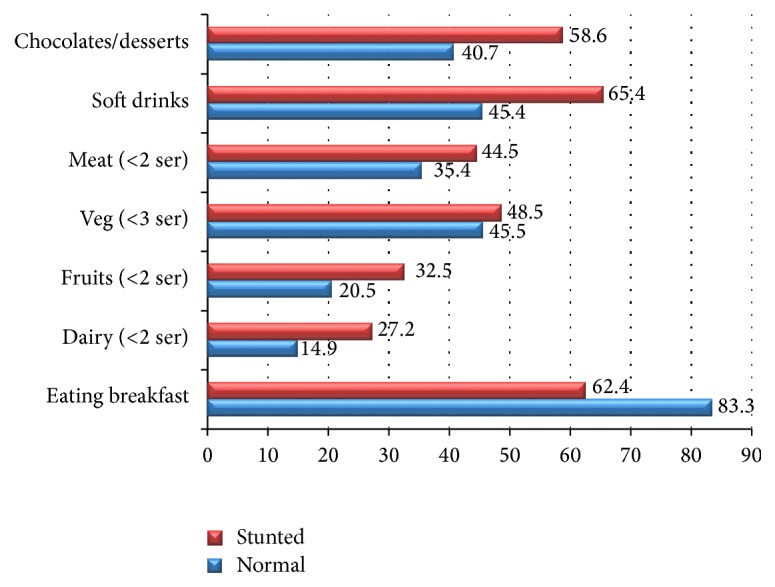
Food and dietary habits of stunted versus normal children and adolescents (2–18 years).

**Figure 2:**
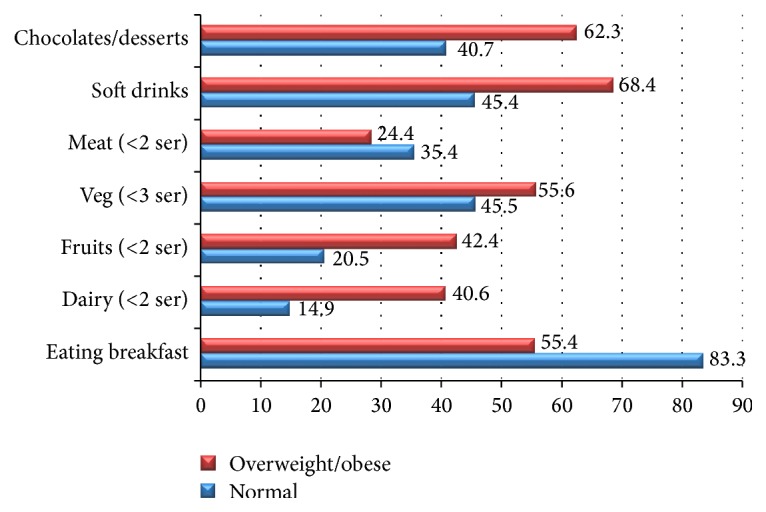
Food and dietary habits of overweight/obese versus other children and adolescents (2–18 years).

Figures 1 and 2 represent food and dietary habits of the stunted and overweight/obese children and adolescents as compared to normal children and adolescents. There is a significant difference (*P* < 0.01) between normal and stunted children and adolescents for various dietary practices like breakfast habit (83.3 versus 62.4%); <2 servings of dairy intake (14.9 versus 27.2%); <2 servings of fruits (20.5 versus 32.5%); soft drinks consumption (45.4 versus 65.4%); and chocolate/sweets/desserts consumption (40.7 versus 58.6%). Similarly, significant differences (*P* < 0.01) were found between overweight/obese and normal children and adolescents for various dietary practices like breakfast habit (83.3 versus 55.4%); <2 servings of dairy intake (14.9 versus 40.6%); <2 servings of fruits (20.5 versus 42.4%); soft drinks consumption (45.4 versus 68.4%); and chocolate/sweets/desserts consumption (40.7 versus 62.3%).

**Figure 3:**
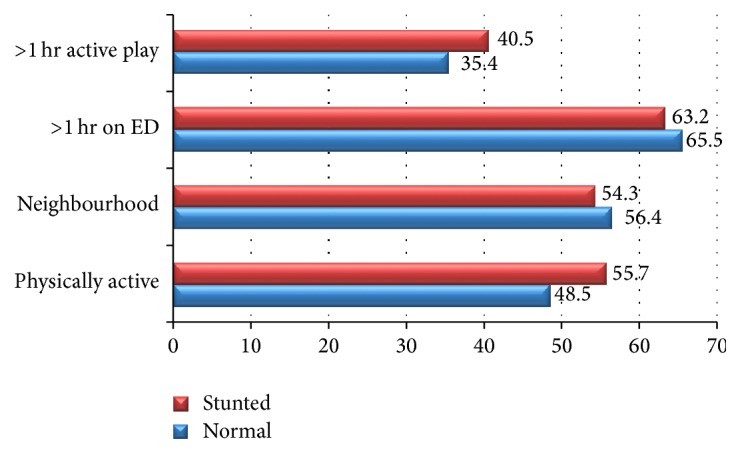
Physical activity (ED^*∗*^) habits of stunted versus normal children and adolescents (2–18 years) (^*∗*^Electronic Devices).

**Figure 4:**
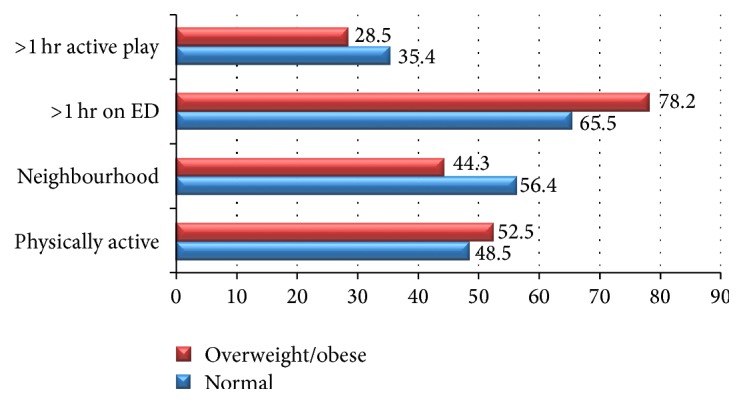
Physical activity (ED^*∗*^) habits of overweight/obese versus normal children and adolescents (2–18 years) (^*∗*^Electronic Devices).

Figures 3 and 4 represent physical activity habits of the stunted and overweight/obese children as compared to normal children and adolescents. There were no statistically significant differences between stunted and normal children and adolescents for physical activity habits. Though, there is a weak trend suggestive of slightly higher percentage of physical activeness among stunted group. However, significant differences (*P* < 0.01) were found between normal and overweight/obese children and adolescents for various physical active habit practices like > 1 hr active play or games (35.4 versus 28.5%); > 1 hr on electronic devices (ED) like computers, laptops, or mobiles (65.5 versus 78.2%); and neighborhood opportunities for playing like availability of parks or open spaces (56.4 versus 44.3%).

Malnutrition in children and adolescents is a serious concern for today's challenging world. Young children, who are at risk for optimal nutrition, have health and survival consequences and, in addition, suffer from physical and intellectual growth which when accumulated can cost ultimately national economic growth [15]. In addition, there is strong evidence that impaired growth is associated with delayed mental development, poor school performance, and reduced intellectual capacity [16, 17] underscoring the need for more increased investment in nutritional interventions [18].

Growth assessment of children as measured through height and weight is the most common method of assessment of prevalence of malnutrition. It is compared to age and gender based growth standards/references to understand the various manifestations of malnutrition. Stunting reflects chronic undernutrition during the most critical periods of growth and development in early life. Wasting reflects acute undernutrition. Underweight is a composite form of undernutrition that includes elements of stunting and wasting [9]. The release of the new WHO child growth standards in 2006 and growth references for ages 5–19 years in 2007, based on selected multinational samples of children with minimal constraints to growth, provided a better standard for comparison for all countries. Understanding the prevalence and patterns of malnutrition in children and adolescents is crucial for any country's planning and implementation of public health policy. Growth monitoring can thus be a powerful tool for decision-making in regard to interventions, to improve physical and psychological growth and development of children. The chief goal is to prevent these vulnerable populations from failing during critical and sensitive periods, which is considerably more costly to treat and have long term implications on their adult health life.

Available research focusing on prevalence of malnutrition in Saudi children is limited. Although one would expect mere absence of undernutrition in Saudi Arabia due to its economic development and resources, in reality comparison of the prevalence of nutritional indicators with selected countries demonstrates only an intermediate position for KSA [10, 19]. WHO country profile page for Saudi Arabia [20] indicates paradigm shift in the prevalence of both wasting and obesity under 5 year children between 1994 (2.9 and 1.2%, resp.) and 2005 (11.8 and 6.1%, resp.) suggesting the existence of double burden of both under- and overnutrition. Previous studies from KSA also confirmed the presence of double burden of malnutrition, that is, presence of both under- and overnutrition among children and adolescents [10–12, 21]. Limited studies available on prevalence of child malnutrition from KSA suggest national data may alone be misleading in understanding the determinants and consequences and stoutly emphasize that it is crucial to focus on regional inequality which can be a result of ethnic as well as environmental conditions [10, 12, 19]. Few available studies documented the existence of prevalence of deficiencies of micronutrients like vitamin A, vitamin D, calcium, iron, iodine, and zinc among children of KSA (all mentioned nutrients known to influence growth and development of children) and also emphasized the need for higher number of studies to understand the wider impact of these deficiencies [22–25].

Present study is in agreement with the prevalence of all forms of malnutrition in the study population, that is, underweight, wasting, stunting, and overweight/obesity indicating there is a need for further exploration of underlying factors, which can explain the key reasons. Although socioeconomic conditions are known to strongly associate with prevalence of undernutrition as indicated in many previous studies from Africa, the region which is still struggling with majority of stunted child population [26], we could not establish any such associations for income, parental education, or birth order in our study.  Table 7 presents the socioeconomic factors associated with prevalence of stunting and wasting. Statistically no significant differences were found. However there was an overall trend towards higher percentage of stunting and wasting with least parent education and income but a higher percentage of overweight/obese with increasing socioeconomic conditions. A higher sample size would have been helpful in identifying gender differences. Since the overall stunted and wasted children prevalence was low, a combined gender analysis was done.

However, our study identified significant dietary and physical activity behaviors which all when mapped together could provide further understanding of factors responsible for nutrition transition effects in KSA.

The overall prevalence of stunting in the present study was found to be around 6.65% and 2.59% for moderate and severe stunting, respectively. Although direct comparisons are not possible between various studies from neighboring regions or even from KSA because of usage of different standards/references, the present study results indicate regional variation in the prevalence rates of stunting as compared to national prevalence of 11%. The study results suggest lower prevalence of stunting as compared to El Mouzan et al. (2012) results for northern region of KSA. In the present study, only Hail region was considered, while the El Mouzan et al. (2012) study calculated prevalence data representing three regions from the north (Hail, Jouf, and Northern Borders). This further suggests within region wise differences.

Wide differences observed for dietary and lifestyle factors between stunted and overweight/obese children as compared to normal children in the present study indicate the complex nutrition, environment interactions, and their effects on growth of the children over a period of time effecting at various stages of life. Rapid urbanization and changing consumption patterns are shifting towards higher energy density, processed foods, including refined grains, and foods higher in saturated fat, sugar, and salt [27]. The study results indicate higher consumption of soft drinks and junk food even among normal children while low intake of protective foods like dairy products and vegetable and fruit consumption is observed in both stunted and overweight/obese children which could be the possible reason for significantly low mean intakes for critical micronutrients which are essential for growth in both groups as compared to normal children. Although the present study calculated the nutrient intake based on 24 hr recall method which could be biased towards over- or underreporting of micronutrient intake, the overall trend across all age and sex groups was indicative of widespread subclinical micronutrient deficiencies. Research is in support of the view that the initial nutritional insults during the early childhood lead to adaptations that may result in obesity during later life [28]. This may partly explain the predominance of stunting in early childhood with progressive increase of overweight and obesity prevalence during adolescence in our study.

The study results are also indicative of higher risk for overweight/obesity among females as compared to males. The physiological reason for obesogenic young females can be simplified for a tipping imbalance between energy consumption and energy expenditure. However, the societal, cultural, behavioral, and environmental conditions that promote and contribute to that imbalance can be too complex and interdependent [29–33]. In addition to being exposed to high calorie low nutrient density foods, females may be additionally vulnerable because of disproportionate opportunities for physical activity in both schools and neighborhood. A recent study which examined whether socioeconomic and behavioral factors may mediate the association between sex and obesity in the Saudi Arabian setting opined that a complex interplay of social and cultural possibly could be the reason for higher obesity prevalence in females in KSA [34]. The present study results further highlight the importance of understanding the mechanisms producing the female excess in obesity even from young age which can help preventing it as early as possible.

## 4. Conclusions

To conclude, this study identifies the prevalence of double burden of malnutrition among all age groups from 2 to 18 years. Stunting decreased with increasing age while overweight/obesity increased with increasing age with females being more vulnerable for the latter. Persisting prevalence of stunting, wasting, and underweight at young ages suggests need for further exploration of dietary practices and environmental influences. Low mean intakes of critical micronutrients among vulnerable groups indicate the need for adequate interventions to address food insecurity and undernutrition. At the same time increasing prevalence of overweight and obesity at later ages from the same region with further deterioration in micronutrient intakes raises red flags and suggests the need for implementation of a multifaceted policy in facing changing nutrition transition challenges. A complex interaction between dietary, nutrition, physical activity, and environment exists and needs further exploration which can provide effective intervention strategies that can address the double-pronged problem of malnutrition.

## Supplementary Material

Designed questionnaires are available online as supplementary file. They were developed based on the various scales available for each of the domains. The major source for the idea of the study design was adopted from the book *Design of the National Children's Study: A Workshop Summary*, National Academics Press, USA, 2013.

## Figures and Tables

**Table 1:** Prevalence of underweight by age and gender.

Age (years)	Total number (%) < −2 SD			Total number (%) < −3 SD		
	Boys	Girls	Combined	Boys	Girls	Combined
2 to <3	48 (5.8)	58 (4.2)	106 (5.0)	48 (1.2)	58 (1.2)	106 (1.2)
3 to <4	55 (4.9)	52 (5.3)	106 (5.1)	55 (2.4)	52 (1.3)	106 (1.85)
4 to <5	47 (7.3)	53 (8.1)	100 (7.7)	47 (1.8)	53 (1.4)	100 (1.6)
5 to 12	225 (3.4)	245 (4.5)	470 (3.95)	225 (2.1)	245 (1.3)	470 (1.7)
13 to 18	325 (2.4)	312 (1.4)	637 (1.9)	325 (1.6)	312 (1.1)	637 (1.35)

*Overall*	*700 (4.76)*	*720 (4.7)*	*1420 (4.73)*	*700 (1.82)*	*720 (1.26)*	*1420 (1.54)*

**Table 2:** Prevalence of stunting by age and gender.

Age (years)	Total number (%) < −2 SD			Total number (%) < −3 SD		
	Boys	Girls	Combined	Boys	Girls	Combined
2 to <3	48 (7.9)	58 (6.4)	106 (7.15)	48 (3.6)	58 (2.4)	106 (3.0)
3 to < 4	55 (8.9)	52 (9.3)	106 (9.1)	55 (3.9)	52 (2.7)	106 (3.3)
4 to <5	47 (8.3)	53 (9.1)	100 (8.7)	47 (3.7)	53 (3.4)	100 (3.55)
5 to 12	225 (6.4)	245 (3.4)	470 (4.9)	225 (2.4)	245 (1.2)	470 (1.8)
13 to 18	325 (2.4)	312 (4.4)	637 (3.4)	325 (1.1)	312 (1.5)	637 (1.3)

*Overall*	*700 (6.78)*	*720 (6.52)*	*1420 (6.65)*	*700 (2.94)*	*720 (2.24)*	*1420 (2.59)*

**Table 3:** Prevalence of wasting by age and gender.

Age (years)	Total number (%) < −2 SD			Total number (%) < −3 SD		
	Boys	Girls	Combined	Boys	Girls	Combined
2 to <3	48 (8.9)	58 (6.8)	106 (7.85)	48 (4.3)	58 (3.4)	106 (3.85)
3 to <4	55 (7.3)	52 (6.4)	106 (6.85)	55 (3.8)	52 (2.7)	106 (3.25)
4 to <5	47 (7.9)	53 (5.9)	100 (6.9)	47 (2.4)	53 (1.4)	100 (1.9)
5 to 12	225 (5.6)	245 (4.5)	470 (5.05)	225 (1.9)	245 (1.7)	470 (1.8)
13 to 18	325 (4.7)	312 (5.4)	637 (5.05)	325 (1.5)	312 (2.4)	637 (1.95)

*Overall*	*700 (6.88)*	*720 (5.8)*	*1420 (6.34)*	*700 (2.78)*	*720 (2.32)*	*1420 (2.55)*

**Table 4:** Prevalence of overweight and obesity by age and gender.

Age (years)	Total number (%) BMI > +1 SD				Total number (%) BMI > +2 SD		
	Boys	Girls		Combined	Boys	Girls	Combined
5 to 12	225 (17.6)	245 (19.5)		470 (18.55)	225 (7.7)	245 (9.7)	470 (8.7)
13 to 18	325 (21.7)	312 (24.4)		637 (23.05)	325 (12.3)	312 (15.4)	637 (13.85)

*Overall*	*700 (19.65)*		*720 (21.95)*	*1420 (20.8)*	*700 (10.0)*	*720 (12.55)*	*1420 (11.27)*

**Table 5:** Mean daily nutrient intake differences between stunted and normal children and adolescents (2–18 years).

Nutrients (per day)	Stunted children	Normal children
Energy (K cal)	894.5 ± 65.9	1043.9 ± 45.7
Protein (g)	37.3 ± 2.3	45.6 ± 4.5
Vitamin A, *μ*g RE	386.8 ± 33.5	427.9 ± 25.8
Thiamine (*μ*g)	0.6 ± 0.02	0.6 ± 0.01
Riboflavin (*μ*g)	0.7 ± 0.03	0.7 ± 0.02
Calcium (mg)	424.7 ± 29.3	553.5 ± 19.4
Iron (mg)	6.1 ± 0.2	7.9 ± 0.5
Zinc (mg)	4.8 ± 0.2	5.2 ± 0.1
Potassium (mg)	1431.0 ± 52.6	1599.1 ± 29.7

**Table 6:** Mean daily nutrient intake differences between overweight/obese and normal children and adolescents (2–18 years).

Nutrients (per day)	Overweight/obese	Normal children
Energy (K cal)	1543.7 ± 105.7	1043.9 ± 45.7
Protein (g)	52.4 ± 7.8	45.6 ± 4.5
Vitamin A, *μ*g RE	377.6 ± 55.3	427.9 ± 25.8
Thiamine (*μ*g)	0.5 ± 0.02	0.6 ± 0.01
Riboflavin (*μ*g)	0.61 ± 0.01	0.7 ± 0.02
Calcium (mg)	363.5 ± 55.7	553.5 ± 19.4
Iron (mg)	8.4 ± 0.24	7.9 ± 0.5
Zinc (mg)	5.7 ± 0.2	5.2 ± 0.1
Potassium (mg)	1386.9 ± 45.6	1599.1 ± 29.7

**Table 7:** Socioeconomic factors associated with prevalence of stunting, wasting, and overweight/obese children (%).

Socioeconomic factors		Stunted children	Wasted children	Overweight/obese children
Mother's education	Illiterate	4.3	3.3	12.3
	Primary/secondary	2.8	3.0	10.7
	University	2.2	2.5	9.4

Father's education	Illiterate	3.4	3.8	9.4
	Primary/secondary	3.7	2.9	11.6
	University	2.2	2.1	12.4

Family income	<5000 SAR	3.7	3.4	9.9
	5000–10000 SAR	3.2	2.7	10.3
	>10000 SAR	2.4	2.7	11.2
