# Supplementary material for: Growth Profile and Its Association with Nutrient Intake and Dietary Patterns among Children and Adolescents in Hail Region of Saudi Arabia
Source: Biomed Res Int. 2017 Feb 20;2017:5740851. doi: 10.1155/2017/5740851 (PMC5337866; doi:10.1155/2017/5740851)
Supplement: Supplementary file 1 — Designed questionnaires are available online as supplementary file. They were developed based on the various scales available for each of the domains. The major source for the idea of the study design was adopted from the book Design of the National Children's Study: A Workshop Summary, National Academics Press, USA, 2013. [file 5740851.f1.doc]

**S1 Questionnaire - Adolescent Boys**

**Questionnaire for boys (13-18 yrs)**

| 1 | Name of the Child: |  | | | |
| --- | --- | --- | --- | --- | --- |
| 2 | Child ID |  | | | |
| 3 | Gender | Male / Female | | | |
| 4 | Date of Birth (DD/MM/YYYY) |  | | | |
| 5 | Height: |  | | | |
| 6 | Weight: |  | | | |
| 7 | Birth Weight |  | | Weight at one yr (If student knows) | |
| 8 | Mid-arm circumference |  | | | |
| 9 | Wrist Circumference |  | | | |
| 10 | Triceps skinfold |  | | | |
| 11 | Subscapular skinfold |  | | | |
| 12 | Calf circumference skinfold |  | | | |
| 13 | Waist circumference |  | | | |
| 14 | Hip Circumference |  | | | |
| 15 | Family Income: | <5000SR= | 5000 –10,000SR= | | >10,000SR= |
| 16 | Father’s Education: | Illiterate= | School Level= | | Graduate= |
| 17 | Mother’’s Education: | Illiterate= | School Level= | | Graduate= |
| 18 | Family Size |  | Order of the child | |  |
| 19 | During the past 6 months did you visit Doctor for minor illnesses | Yes= | No= | | If yes, how many visits and reason for visit |
| 20 | Do you suffer from frequent cold /cough/ fever | Yes= | No= | | If yes, is the child treated with antibiotics frequently  Yes/ No |

| **Medical History** | **Yes** | **No** | **If yes, when noticed** | **Currently under Treatment** | **Any other info** |
| --- | --- | --- | --- | --- | --- |
| 1. Sight problems |  |  |  |  |  |
| 1. Hearing problems |  |  |  |  |  |
| 1. Speech problems |  |  |  |  |  |
| 1. Dental Problems |  |  |  |  |  |
| 1. Blackouts, fits or loss of consciousness |  |  |  |  |  |
| 1. Difficulty learning or understanding things |  |  |  |  |  |
| 1. Nerves or emotional conditions that need treatment |  |  |  |  |  |
| 1. Any disfigurement or deformity (eg: bow legs) |  |  |  |  |  |
| 1. Difficulty in breathing or known pulmonary conditions (Asthma, Bronchitis etc.) |  |  |  |  |  |
| 1. Sleep Apnea/ sleep disorders |  |  |  |  |  |
| 1. Anaemia |  |  |  |  |  |
| 1. Frequent gastrointestinal disturbances |  |  |  |  |  |
| 1. Chronic or recurring pain |  |  |  |  |  |
| 1. Ulcers |  |  |  |  |  |
| 1. Edema (current pitting edema/ history) |  |  |  |  |  |
| 1. Appetite /Eating problems — eats poorly or too much, etc |  |  |  |  |  |
| 1. Diabetes |  |  |  |  |  |
| 1. Hypertension |  |  |  |  |  |
| 1. Hyperlipidemia |  |  |  |  |  |
| 1. Any other, mention details |  |  |  |  |  |

| **Food habits and Lifestyle History /** | **Yes** | **No** | **If yes, how frequently**  **(Regularly, most times, occasionally)** | **Any other info** |
| --- | --- | --- | --- | --- |
| 1. Are you on special diet |  |  |  |  |
| 1. Are you allergic to any foods |  |  |  |  |
| 1. Do you eat non food items like clay, mud, paint etc |  |  |  |  |
| 1. Do you drink 1 ½ cup or more of fruit drinks a day |  |  |  |  |
| 1. Do you drink coke, Kool -Aids, sodas |  |  |  |  |
| 1. Do you like drinking plain milk |  |  |  |  |
| 1. Do you like drinking plain yogurt or Laban |  |  |  |  |
| 1. Do you drink flavoured milks regularly |  |  |  |  |
| 1. Do you take flavoured yogurts regularly |  |  |  |  |
| 1. Do you like eating raw vegetables |  |  |  |  |
| 1. Do you like eating green leafy vegetables |  |  |  |  |
| 1. Do you like eating fruits |  |  |  |  |
| 1. Do you like eating chips |  |  |  |  |
| 1. Do you like eating chocolates |  |  |  |  |
| 1. Do you like to eat in restaurants |  |  |  |  |
| 1. Do you like fast foods |  |  |  |  |
| 1. Do you eat together with your family |  |  |  |  |
| 1. How many days in a week you eat outside the home with friends |  |  |  |  |
| 1. Do you eat breakfast regularly |  |  |  |  |
| 1. Do you skip your meals |  |  |  |  |
| 1. Do you smoke |  |  |  |  |
| 1. Are you currently using any multivitamin or mineral supplements |  |  |  |  |
| 1. Are you using any protein supplements |  |  |  |  |
| 1. Are you currently practising dieting |  |  |  |  |

| **Diet history** | **Options** | **Any other info** |
| --- | --- | --- |
| 1. What is the main type of milk given to you | Whole milk/ skim milk/ evaporated milk/ soya milk/ doesn’t drink milk/ other (specify) |  |
| 1. How many cups of milk /yogurt you drink everyday | Less than one cup/ 1/2/3/4 or more |  |
| 1. How many serves of cheese you eat everyday (**One serve is equal to 1 ounce/ 1 slice)** | Less than one serve/ 1/2/3/4 or more |  |
| 1. How many serves of cooked vegetables you eat each day? **One serve is equal to half a cup.** | Less than one serve/ 1/2/3/4 or more |  |
| 1. How many serves of raw vegetables you eat each day? **One serve is equal to one cup.** | Less than one serve/ 1/2/3/4 or more |  |
| 1. How many serves of fruits you eat each day? **One serve is equal to half a cup of chopped fruits or one medium sized fruit.** | Less than one serve/ 1/2/3/4 or more |  |
| 1. How many serves of meat/egg/chicken/beans you eat daily? **(One serve is equal to half egg/one ounce of meat/half cup cooked beans)** | Less than one serve/ 1/2/3/4 or more |  |
| 1. How many serves of bread /rice you eat daily **(1 slice of bread/1/2 cup of dry cereal/ ½ cup of cooked rice/pasta)** | Less than one serve/ 1/2/3/4/5/6/or more |  |
| 1. Is iodised salt used for cooking usually | Yes/ no / Sometimes |  |
| 1. Do you add salt at table usually | Yes/ no / Sometimes |  |

| **Developmental Mile stones** | **Yes** | **No** |
| --- | --- | --- |
| 1. Is there a change in your voice |  |  |
| 1. Do you have good relationship with your friends |  |  |
| 1. Can you perform all self cores with out depending on your parents |  |  |
| 1. Do you have acne |  |  |

| **Physical Activity /Neighbourhood** | **Options** |
| --- | --- |
| 1. What is the main type of games played by the child | Running/ playing with friends/ electronic games/ riding bicycle/ skating/ football/ swimming/others(specify) |
| 1. How often you go to park to play | Daily / Weekly one to two times/ Weekly three- five times/ Other (specify) |
| 1. Do you have enough space for playing in your backyard | Yes / No |
| 1. Do you have enough area to ride a bicycle in your neighbourhood | Yes/No |
| 1. Does your local neighbourhood have the following places or facilities where you can play and be physically active? | Open areas / Public park or Playground/ swimming pool /Gym /Club that offers activities/sports |
| 1. No. of hours you spend in watching TV daily |  |
| 1. No. of hours you spend in playing electronic games daily |  |
| 1. No. of hours you spend with computer |  |
| 1. How do you go to school daily | Walking/ car or bus |
| 1. How many hrs / mins you play in school daily |  |

**24 hr Recall Diet History**

| **Meal Time** | **Time** | **Food Item along with preparation method** | **Serving size** | **Place of Consumption** |
| --- | --- | --- | --- | --- |
| **Morning** |  |  |  |  |
| **Mid morning** |  |  |  |  |
| **Lunch** |  |  |  |  |
| **Evening Snacks** |  |  |  |  |
| **Dinner** |  |  |  |  |
|  |  |  |  |  |

**S2 Questionnaire - Adolescent Girls**

**Questionnaire for girls (11-18 yrs)**

| 1 | Name of the Child: |  | | | |
| --- | --- | --- | --- | --- | --- |
| 2 | Child ID |  | | | |
| 3 | Gender | Male / Female | | | |
| 4 | Date of Birth (DD/MM/YYYY) |  | | | |
| 5 | Height: |  | | | |
| 6 | Weight: |  | | | |
| 7 | Birth Weight |  | | Weight at one yr (If student knows) | |
| 8 | Mid-arm circumference |  | | | |
| 9 | Wrist Circumference |  | | | |
| 10 | Triceps skinfold |  | | | |
| 11 | Subscapular skinfold |  | | | |
| 12 | Calf circumference skinfold |  | | | |
| 13 | Waist circumference |  | | | |
| 14 | Hip Circumference |  | | | |
| 15 | Family Income: | <5000SR= | 5000 –10,000SR= | | >10,000SR= |
| 16 | Father’s Education: | Illiterate= | School Level= | | Graduate= |
| 17 | Mother’’s Education: | Illiterate= | School Level= | | Graduate= |
| 18 | Family Size |  | Order of the child | |  |
| 19 | During the past 6 months did you visit Doctor for minor illnesses | Yes= | No= | | If yes, how many visits and reason for visit |
| 20 | Do you suffer from frequent cold /cough/ fever | Yes= | No= | | If yes, is the child treated with antibiotics frequently  Yes/ No |

| **Medical History** | **Yes** | **No** | **If yes, when noticed** | **Currently under Treatment** | **Any other info** |
| --- | --- | --- | --- | --- | --- |
| 1. Sight problems |  |  |  |  |  |
| 1. Hearing problems |  |  |  |  |  |
| 1. Speech problems |  |  |  |  |  |
| 1. Dental Problems |  |  |  |  |  |
| 1. Blackouts, fits or loss of consciousness |  |  |  |  |  |
| 1. Difficulty learning or understanding things |  |  |  |  |  |
| 1. Nerves or emotional conditions that need treatment |  |  |  |  |  |
| 1. Any disfigurement or deformity (eg: bow legs) |  |  |  |  |  |
| 1. Difficulty in breathing or known pulmonary conditions (Asthma, Bronchitis etc.) |  |  |  |  |  |
| 1. Sleep Apnea/ sleep disorders |  |  |  |  |  |
| 1. Anaemia |  |  |  |  |  |
| 1. Frequent gastrointestinal disturbances |  |  |  |  |  |
| 1. Chronic or recurring pain |  |  |  |  |  |
| 1. Ulcers |  |  |  |  |  |
| 1. Edema (current pitting edema/ history) |  |  |  |  |  |
| 1. Appetite /Eating problems — eats poorly or too much, etc |  |  |  |  |  |
| 1. Diabetes |  |  |  |  |  |
| 1. Hypertension |  |  |  |  |  |
| 1. Hyperlipidemia |  |  |  |  |  |
| 1. Any other, mention details |  |  |  |  |  |

| **Food habits and Lifestyle History /** | **Yes** | **No** | **If yes, how frequently**  **(Regularly, most times, occasionally)** | **Any other info** |
| --- | --- | --- | --- | --- |
| 1. Are you on special diet |  |  |  |  |
| 1. Are you allergic to any foods |  |  |  |  |
| 1. Do you eat non food items like clay, mud, paint etc |  |  |  |  |
| 1. Do you drink 1 ½ cup or more of fruit drinks a day |  |  |  |  |
| 1. Do you drink coke, Kool -Aids, sodas |  |  |  |  |
| 1. Do you like drinking plain milk |  |  |  |  |
| 1. Do you like drinking plain yogurt or Laban |  |  |  |  |
| 1. Do you drink flavoured milks regularly |  |  |  |  |
| 1. Do you take flavoured yogurts regularly |  |  |  |  |
| 1. Do you like eating raw vegetables |  |  |  |  |
| 1. Do you like eating green leafy vegetables |  |  |  |  |
| 1. Do you like eating fruits |  |  |  |  |
| 1. Do you like eating chips |  |  |  |  |
| 1. Do you like eating chocolates |  |  |  |  |
| 1. Do you like to eat in restaurants |  |  |  |  |
| 1. Do you like fast foods |  |  |  |  |
| 1. Do you eat together with your family |  |  |  |  |
| 1. How many days in a week you eat outside the home with friends |  |  |  |  |
| 1. Do you eat breakfast regularly |  |  |  |  |
| 1. Do you skip your meals |  |  |  |  |
| 1. Do you smoke |  |  |  |  |
| 1. Are you currently using any multivitamin or mineral supplements |  |  |  |  |
| 1. Are you using any protein supplements |  |  |  |  |
| 1. Are you currently practising dieting |  |  |  |  |

| **Diet history** | **Options** | **Any other info** |
| --- | --- | --- |
| 1. What is the main type of milk given to you | Whole milk/ skim milk/ evaporated milk/ soya milk/ doesn’t drink milk/ other (specify) |  |
| 1. How many cups of milk /yogurt you drink everyday | Less than one cup/ 1/2/3/4 or more |  |
| 1. How many serves of cheese you eat everyday (**One serve is equal to 1 ounce/ 1 slice)** | Less than one serve/ 1/2/3/4 or more |  |
| 1. How many serves of cooked vegetables you eat each day? **One serve is equal to half a cup.** | Less than one serve/ 1/2/3/4 or more |  |
| 1. How many serves of raw vegetables you eat each day? **One serve is equal to one cup.** | Less than one serve/ 1/2/3/4 or more |  |
| 1. How many serves of fruits you eat each day? **One serve is equal to half a cup of chopped fruits or one medium sized fruit.** | Less than one serve/ 1/2/3/4 or more |  |
| 1. How many serves of meat/egg/chicken/beans you eat daily? **(One serve is equal to half egg/one ounce of meat/half cup cooked beans)** | Less than one serve/ 1/2/3/4 or more |  |
| 1. How many serves of bread /rice you eat daily **(1 slice of bread/1/2 cup of dry cereal/ ½ cup of cooked rice/pasta)** | Less than one serve/ 1/2/3/4/5/6/or more |  |
| 1. Is iodised salt used for cooking usually | Yes/ no / Sometimes |  |
| 1. Do you add salt at table usually | Yes/ no / Sometimes |  |

| **Developmental Mile stones** | **Yes** | **No** |
| --- | --- | --- |
| 1. Are you currently menstruating. If yes, from which year you had menstruation cycle |  |  |
| 1. Do you experience pain during menstrual cycle |  |  |
| 1. Do you have mood swings during menstrual cycle |  |  |
| 1. Do you have good relationship with your friends |  |  |
| 1. Can you perform all self cores with out depending on your parents |  |  |
| 1. Do you have acne |  |  |

| **Physical Activity /Neighbourhood** | **Options** |
| --- | --- |
| 1. What is the main type of games played by the child | Running/ playing with friends/ electronic games/ riding bicycle/ skating/ football/ swimming/others(specify) |
| 1. How often you go to park to play | Daily / Weekly one to two times/ Weekly three- five times/ Other (specify) |
| 1. Do you have enough space for playing in your backyard | Yes / No |
| 1. Do you have enough area to ride a bicycle in your neighbourhood | Yes/No |
| 1. Does your local neighbourhood have the following places or facilities where you can play and be physically active? | Open areas / Public park or Playground/ swimming pool /Gym /Club that offers activities/sports |
| 1. No. of hours you spend in watching TV daily |  |
| 1. No. of hours you spend in playing electronic games daily |  |
| 1. No. of hours you spend with computer |  |
| 1. How do you go to school daily | Walking/ car or bus |
| 1. How many hrs / mins you play in school daily |  |

**24 hr Recall Diet History**

| **Meal Time** | **Time** | **Food Item along with preparation method** | **Serving size** | **Place of Consumption** |
| --- | --- | --- | --- | --- |
| **Morning** |  |  |  |  |
| **Mid morning** |  |  |  |  |
| **Lunch** |  |  |  |  |
| **Evening Snacks** |  |  |  |  |
| **Dinner** |  |  |  |  |
|  |  |  |  |  |

**S3 Questionnaire - Boys & Girls- 2- 5 yrs**

**Questionnaire for boys and girls – 2-5 yrs**

| 1 | Name of the Child: |  | | | | |
| --- | --- | --- | --- | --- | --- | --- |
| 2 | Child ID |  | | | | |
| 3 | Gender | Male / Female | | | | |
| 4 | Date of Birth (DD/MM/YYYY) |  | | | | |
| 5 | Height: |  | | | | |
| 6 | Weight: |  | | | | |
| 7 | Birth Weight |  | | Weight at one yr (If parents remember) | | |
| 8 | Head circumference |  | | | | |
| 9 | Mid-arm circumference |  | | | | |
| 10 | Wrist Circumference |  | | | | |
| 11 | Calf circumference |  | | | | |
| 12 | Triceps skinfold |  | | | | |
| 13 | Subscapular skinfold |  | | | | |
| 14 | Calf skinfold |  | | | | |
| 15 | Family Income: | <5000SR= | 5000 –10,000SR= | | >10,000SR= | |
| 16 | Father’s Education: | Illiterate= | School Level= | | Graduate= | |
| 17 | Mother’s Education: | Illiterate= | School Level= | | Graduate= | |
| 18 | Father Working | Yes= | No= | | Name of the Occupation: | |
| 19 | Mother Working | Yes= | No= | | Name of the Occupation: | |
| 20 | Family Size |  | Order of the child | |  | |
| 21 | Mode of delivery of the study child | Normal | Caesarean | | Forceps/Vacuum extraction | |
| 22 | Any complications to mother during delivery of the study child | Preeclampsia/  Hypertension | Gestational diabetes | | Thyroid disorders | Any other (give details) |
| 23 | Breast fed | Yes= | No= | | Age till = | Currently breast feeding |
| 24 | Bottle feeding | Yes= | No= | | Age till = | Simultaneously along with Breast feeding  Yes= / No= |
| 25 | Age at which weaning introduced | |  | | | |
| 26 | Age at which solid foods introduced | |  | | | |
| 27 | Age at which egg/meat/products introduced | |  | | | |
| 28 | Age at which soft drinks/colas introduced | |  | | | |
| 29 | Vaccinations regularly given | Yes= | No= | | If no, reason | |
| 30 | During the past 6 months did the child visit Doctor for minor illnesses | Yes= | No= | | If yes, how many visits and reason for visit | |
| 31 | Does child suffer from frequent cold /cough/ fever | Yes= | No= | | If yes, is the child treated with antibiotics frequently  Yes/ No | |
| 32 | Does child suffer frequent diarrhoea/vomitings | Yes= | No= | | Possible Reasons | |

| **Medical History** | **Yes** | **No** | **If yes, age when noticed** | **Currently under Treatment** | **Any other info** |
| --- | --- | --- | --- | --- | --- |
| 1. Sight problems |  |  |  |  |  |
| 1. Hearing problems |  |  |  |  |  |
| 1. Speech problems |  |  |  |  |  |
| 1. Dental Problems |  |  |  |  |  |
| 1. Blackouts, fits or loss of consciousness |  |  |  |  |  |
| 1. Difficulty learning or understanding things |  |  |  |  |  |
| 1. Limited use of arms or fingers |  |  |  |  |  |
| 1. Difficulty gripping things |  |  |  |  |  |
| 1. Limited use of legs or feet |  |  |  |  |  |
| 1. Nerves or emotional conditions that need treatment |  |  |  |  |  |
| 1. Any disfigurement or deformity (eg: bow legs) |  |  |  |  |  |
| 1. Difficulty in breathing or known pulmonary conditions (Asthma, Bronchitis etc.) |  |  |  |  |  |
| 1. Sleep Apnea |  |  |  |  |  |
| 1. Anaemia |  |  |  |  |  |
| 1. Frequent gastrointestinal disturbances |  |  |  |  |  |
| 1. Chronic or recurring pain |  |  |  |  |  |
| 1. Any condition that restricts physical activity or physical work |  |  |  |  |  |
| 1. Edema (current pitting edema/ history) |  |  |  |  |  |
| 1. Eating problems — eats poorly or too much, etc |  |  |  |  |  |
| 1. Bowel and bladder problems, toilet training |  |  |  |  |  |

| **Food habits History /** | **Yes** | **No** | **Reasons / details** | **Any other info** |
| --- | --- | --- | --- | --- |
| 1. Is the child on special diet |  |  |  |  |
| 1. Is the child allergic to any foods |  |  |  |  |
| 1. Is the child still on breast feed |  |  |  |  |
| 1. Is the child still on bottle feed |  |  |  |  |
| 1. Does the child eat non food items like clay, mud, paint etc |  |  |  |  |
| 1. Does your child drink 1 ½ cup or more of fruit drinks a day |  |  |  |  |
| 1. Does your child drink fruit drinks, Kool -Aids, sodas |  |  |  |  |
| 1. Does the child given non-fat or reduced fat milk before 2 years |  |  |  |  |
| 1. Does the child like eating raw vegetables |  |  |  |  |
| 1. Does the child like eating green leafy vegetables |  |  |  |  |
| 1. Does the child like eating fruits |  |  |  |  |
| 1. Does the child like drinking milk |  |  |  |  |
| 1. Does the child eat with his own hands or fed by parents |  |  |  |  |
| 1. Does the family eat together along with the child |  |  |  |  |

| **Diet history** | **Options** | **Any other info** |
| --- | --- | --- |
| 1. What is the main type of milk given to child | Whole milk/ skim milk/ evaporated milk/ soya milk/ doesn’t drink milk/ other (specify) |  |
| 1. How many cups of milk /yogurt does the child drink everyday | Less than one cup/ 1/2/3/4 or more |  |
| 1. How many serves of cheese does the child eat everyday (**One serve is equal to 1 ounce/ 1 slice)** | Less than one serve/ 1/2/3/4 or more |  |
| 1. How many serves of cooked vegetables does the child eat each day? **One serve is equal to half a cup.** | Less than one serve/ 1/2/3/4 or more |  |
| 1. How many serves of raw vegetables does the child eat each day? **One serve is equal to one cup.** | Less than one serve/ 1/2/3/4 or more |  |
| 1. How many serves of fruits does the child eat each day? **One serve is equal to half a cup of chopped fruits or one medium sized fruit.** | Less than one serve/ 1/2/3/4 or more |  |
| 1. How many serves of meat/egg/chicken/fish/beans does the child eat daily? **(One serve is equal to half egg/one ounce of meat/half cup cooked beans)** | Less than one serve/ 1/2/3/4 or more |  |
| 1. How many serves of bread /rice the child eat daily **(1 slice of bread/1/2 cup of dry cereal/ ½ cup of cooked rice/pasta)** | Less than one serve/ 1/2/3/4/5/6/or more |  |
| 1. Is iodised salt used for cooking usually | Yes/ no / Sometimes |  |
| 1. Do you add salt at table usually | Yes/ no / Sometimes |  |
| 1. Does the child eat breakfast regularly | Yes/ no / Sometimes |  |
| 1. If not regular, how many days in a week does the child eat breakfast |  |  |

| **Gross Motor Development** | **Yes** | **No** |
| --- | --- | --- |
| **Age 2-3** | | |
| 1. Runs smoothly, turning corners and making sudden stops. |  |  |
| 1. Climbs up ladder and slides down slide without help. |  |  |
| 1. Jumps from steps with feet together. Or used to. |  |  |
| 1. Stands on one foot for a few seconds without support. |  |  |
| 1. Does a forward somersault |  |  |
| 1. From a standing position, jumps over objects or people |  |  |
| 1. Walks up and down stairs alone, one foot to a step, alternating feet |  |  |
| **Age 3-4** | | |
| 1. When running, jumps over obstacles that are in the way. |  |  |
| 1. Rides around on tricycle using pedals. |  |  |
| 1. Stands on one foot, steady, without support. |  |  |
| 1. Hops on one foot, at least two times, without support. |  |  |
| **Age 4-5** | | |
| 1. Hops around on one foot without support. |  |  |
| 1. Plays “catch” with other children; throwing to them and catching the ball at least half the time. |  |  |
| 1. Swings on swing, pumping by self. |  |  |

| **Fine Motor Development** | **Yes** | **No** |
| --- | --- | --- |
| **Age 2-3** | | |
| 1. Unscrews and screws on covers of jars or bottles |  |  |
| 1. Places single pieces — simple shapes or figures — in a puzzle board. |  |  |
| 1. Attempts to cut with small scissors. Or cuts |  |  |
| 1. Holds crayon with fingers and thumb, somewhat like an adult |  |  |
| 1. Scribbles with a circular motion |  |  |
| **Age 3-4** | | |
| 1. Draws or copies vertical ( | ) and horizontal ( __ ) lines or complete circles |  |  |
| 1. Builds things with blocks, such as a simple house, bridge, or car |  |  |
| 1. Cuts across paper with scissors from one side to the other |  |  |
| 1. Puts together puzzles with nine or more pieces. |  |  |
| **Age 4-5** | | |
| 1. Draws recognizable pictures |  |  |
| 1. Cuts with scissors, following a simple outline or pattern. |  |  |
| 1. Colors within the lines in a coloring book |  |  |

| **Physical Activity /Neighbourhood** | **Options** |
| --- | --- |
| 1. What is the main type of games played by the child | Running/ Solo play with toys/ playing with other kids/ electronic games/ riding tricycle/ other (specify) |
| 1. How often you take the child to park | Daily / Weekly one to two times/ Weekly three- five times/ Other (specify) |
| 1. Do you have enough space for playing for the child in your backyard | Yes / No |
| 1. Do you have enough area suitable for the child to ride a tricycle, bike or scooter etc | Yes/No |
| 1. Does your local neighbourhood have the following places or facilities where your child can be play and be physically active? | Open areas / Public park or Playground/ swimming pool /Gym that offers programs for young children e.g. kindergym, playgym etc / Club that offers activities/sports for young children e.g. soccer, dance etc. |
| 1. Does your child play outdoors in your neighbourhood | Yes/No |
| 1. No. of hours the child spend in watching TV daily |  |
| 1. No. of hours the child spend in playing electronic games daily |  |

**24 hr Recall Diet History**

| **Meal Time** | **Time** | **Food Item along with preparation method** | **Serving size** |
| --- | --- | --- | --- |
| **Morning** |  |  |  |
| **Mid morning** |  |  |  |
| **Lunch** |  |  |  |
| **Evening Snacks** |  |  |  |
| **Dinner** |  |  |  |
|  |  |  |  |

**S4 Questionnaire - Middle School Boys & Girls**

**Questionnaire for boys (6-12 yrs) and girls (6-10 yrs)**

| 1 | Name of the Child: |  | | | | |
| --- | --- | --- | --- | --- | --- | --- |
| 2 | Child ID |  | | | | |
| 3 | Gender | Male / Female | | | | |
| 4 | Date of Birth (DD/MM/YYYY) |  | | | | |
| 5 | Height: |  | | | | |
| 6 | Weight: |  | | | | |
| 7 | Birth Weight |  | | Weight at one yr (If parents remember) | | |
| 8 | Mid-arm circumference |  | | | | |
| 9 | Wrist Circumference |  | | | | |
| 10 | Triceps skinfold |  | | | | |
| 11 | Subscapular skinfold |  | | | | |
| 12 | Calf circumference skinfold |  | | | | |
| 13 | Waist circumference |  | | | | |
| 14 | Hip Circumference |  | | | | |
| 15 | Family Income: | <5000SR= | 5000 –10,000SR= | | >10,000SR= | |
| 16 | Father’s Education: | Illiterate= | School Level= | | Graduate= | |
| 17 | Mother’’s Education: | Illiterate= | School Level= | | Graduate= | |
| 18 | Family Size |  | Order of the child | |  | |
| 19 | Mode of delivery of the study child | Normal | Caesarean | | Forceps/Vacuum extraction | |
| 20 | Any complications to mother during delivery of the study child | Preeclampsia/  Hypertension | Gestational diabetes | | Thyroid disorders | Any other (give details) |
| 21 | Breast fed | Yes= | No= | | Age till = | |
| 24 | Vaccinations regularly given | Yes= | No= | | If no, reason | |
| 25 | During the past 6 months did the child visit Doctor for minor illnesses | Yes= | No= | | If yes, how many visits and reason for visit | |
| 26 | Does child suffer from frequent cold /cough/ fever | Yes= | No= | | If yes, is the child treated with antibiotics frequently  Yes/ No | |
| 27 | Does child suffer frequent diarrhoea/vomitings | Yes= | No= | |  | |
| 30 | Exercise / Physical games /Day | <30min | 30min | | >30min | |

| **Medical History** | **Yes** | **No** | **If yes, when noticed** | **Currently under Treatment** | **Any other info** |
| --- | --- | --- | --- | --- | --- |
| 1. Sight problems |  |  |  |  |  |
| 1. Hearing problems |  |  |  |  |  |
| 1. Speech problems |  |  |  |  |  |
| 1. Dental Problems |  |  |  |  |  |
| 1. Blackouts, fits or loss of consciousness |  |  |  |  |  |
| 1. Difficulty learning or understanding things |  |  |  |  |  |
| 1. Limited use of arms or fingers |  |  |  |  |  |
| 1. Difficulty gripping things |  |  |  |  |  |
| 1. Limited use of legs or feet |  |  |  |  |  |
| 1. Nerves or emotional conditions that need treatment |  |  |  |  |  |
| 1. Any disfigurement or deformity (eg: bow legs) |  |  |  |  |  |
| 1. Difficulty in breathing or known pulmonary conditions (Asthma, Bronchitis etc.) |  |  |  |  |  |
| 1. Sleep Apnea |  |  |  |  |  |
| 1. Anaemia |  |  |  |  |  |
| 1. Frequent gastrointestinal disturbances |  |  |  |  |  |
| 1. Chronic or recurring pain |  |  |  |  |  |
| 1. Any condition that restricts physical activity or physical work |  |  |  |  |  |
| 1. Edema (current pitting edema/ history) |  |  |  |  |  |
| 1. Eating problems — eats poorly or too much, etc |  |  |  |  |  |
| 1. Any other, mention details |  |  |  |  |  |

| **Food habits History /** | **Yes** | **No** | **If yes, how frequently**  **(Regularly, most times, occasionally)** | **Any other info** |
| --- | --- | --- | --- | --- |
| 1. Is the child on special diet |  |  |  |  |
| 1. Is the child allergic to any foods |  |  |  |  |
| 1. Does the child eat non food items like clay, mud, paint etc |  |  |  |  |
| 1. Does the child drink 1 ½ cup or more of fruit drinks a day |  |  |  |  |
| 1. Does the child drink fruit drinks, Kool -Aids, sodas |  |  |  |  |
| 1. Does the child like drinking plain milk |  |  |  |  |
| 1. Does the child like drinking plain yogurt or Laban |  |  |  |  |
| 1. Does the child drink flavoured milks regularly |  |  |  |  |
| 1. Does the child drink flavoured yogurts regularly |  |  |  |  |
| 1. Does the child like eating raw vegetables |  |  |  |  |
| 1. Does the child like eating green leafy vegetables |  |  |  |  |
| 1. Does the child like eating fruits |  |  |  |  |
| 1. Does the child like eating chips |  |  |  |  |
| 1. Does the child like eating chocolates |  |  |  |  |
| 1. Does the child like to eat in restaurants |  |  |  |  |
| 1. Does the child like fast foods |  |  |  |  |
| 1. Does the family eat together along with the child |  |  |  |  |

| **Diet history** | **Options** | **Any other info** |
| --- | --- | --- |
| 1. What is the main type of milk given to child | Whole milk/ skim milk/ evaporated milk/ soya milk/ doesn’t drink milk/ other (specify) |  |
| 1. How many cups of milk /yogurt does the child drink everyday | Less than one cup/ 1/2/3/4 or more |  |
| 1. How many serves of cheese does the child eat everyday (**One serve is equal to 1 ounce/ 1 slice)** | Less than one serve/ 1/2/3/4 or more |  |
| 1. How many serves of cooked vegetables does the child eat each day? **One serve is equal to half a cup.** | Less than one serve/ 1/2/3/4 or more |  |
| 1. How many serves of raw vegetables does the child eat each day? **One serve is equal to one cup.** | Less than one serve/ 1/2/3/4 or more |  |
| 1. How many serves of fruits does the child eat each day? **One serve is equal to half a cup of chopped fruits or one medium sized fruit.** | Less than one serve/ 1/2/3/4 or more |  |
| 1. How many serves of meat/egg/chicken/beans does the child eat daily? **(One serve is equal to half egg/one ounce of meat/half cup cooked beans)** | Less than one serve/ 1/2/3/4 or more |  |
| 1. How many serves of bread /rice the child eat daily **(1 slice of bread/1/2 cup of dry cereal/ ½ cup of cooked rice/pasta)** | Less than one serve/ 1/2/3/4/5/6/or more |  |
| 1. Is iodised salt used for cooking usually | Yes/ no / Sometimes |  |
| 1. Do you add salt at table usually | Yes/ no / Sometimes |  |
| 1. Does the child eat breakfast regularly | Yes/ no / Sometimes |  |
| 1. If not regular, how many days in a week does the child eat breakfast |  |  |

| **Gross and Fine Motor Development** | **Yes** | **No** |
| --- | --- | --- |
| 1. Able to balance intense activities (eg: riding bicycle) |  |  |
| 1. Eruption of permanent teeth |  |  |
| 1. Able to perform self cores like taking bath, brushing, keeping room tidy etc |  |  |
| 1. Able to perform reading, and painting |  |  |
| 1. Ability to complete complex puzzles |  |  |

| **Social and Mental Development** | **Yes** | **No** |
| --- | --- | --- |
| 1. Attention span greater than 15 minutes |  |  |
| 1. Cordial social relationships in school |  |  |
| 1. Ability to express their problems and ability to see through solutions |  |  |
| 1. Mastering sequencing and ordering |  |  |

| **Physical Activity /Neighbourhood** | **Options** |
| --- | --- |
| 1. What is the main type of games played by the child | Running/ Solo play with toys/ playing with other kids/ electronic games/ riding tricycle/ other (specify) |
| 1. How often you go to park to play | Daily / Weekly one to two times/ Weekly three- five times/ Other (specify) |
| 1. Do you have enough space for playing in your backyard | Yes / No |
| 1. Do you have enough area to ride a bicycle in your neighbourhood | Yes/No |
| 1. Does your local neighbourhood have the following places or facilities where you can play and be physically active? | Open areas / Public park or Playground/ swimming pool /Gym /Club that offers activities/sports for young children e.g. soccer, dance etc. |
| 1. No. of hours you spend in watching TV daily |  |
| 1. No. of hours the child spend in playing electronic games daily |  |
| 1. How do you go to school daily | Walking/ car or bus |
| 1. How many hrs / mins you play in school daily |  |

**24 hr Recall Diet History**

| **Meal Time** | **Time** | **Food Item along with preparation method** | **Serving size** |
| --- | --- | --- | --- |
| **Morning** |  |  |  |
| **Mid morning** |  |  |  |
| **Lunch** |  |  |  |
| **Evening Snacks** |  |  |  |
| **Dinner** |  |  |  |
|  |  |  |  |
